# Advances in systemic therapy for advanced pancreatobiliary malignancies

**DOI:** 10.12688/f1000research.2-105.v1

**Published:** 2013-04-08

**Authors:** Thorvardur R Halfdanarson, Sigurdis Haraldsdottir, Mitesh J Borad

**Affiliations:** 1 Division of Hematology and Medical Oncology, Mayo Clinic Arizona, Scottsdale, AZ, USA; 2 The Ohio State University Comprehensive Cancer Center, Columbus, OH, 43210, USA

## Abstract

Pancreatobiliary malignancies are relatively uncommon and the overall prognosis is poor. Treatment options for advanced disease are limited to systemic therapy for metastatic disease and a combination of systemic therapy and radiation therapy for locally advanced but unresectable tumors. There have been significant advances in the treatment of pancreatobiliary cancers in recent years but the prognosis for patient survival remains disappointingly poor. We review the current treatment options for locally advanced pancreatobiliary malignancies and highlight recent advances in systemic therapy, including novel approaches using targeted treatments.

## Introduction

**Figure 1.**
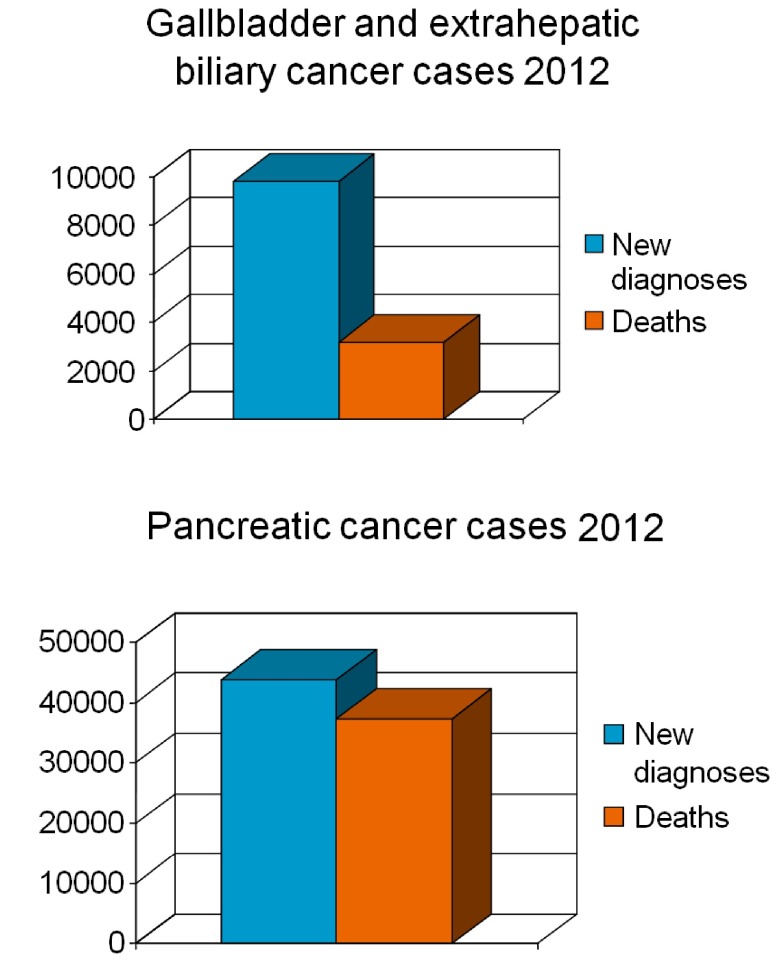
Number of expected new cases and deaths of pancreatic cancer and gallbladder and extrahepatic biliary cancer in the United States in 2012 ^1^.

Pancreatobiliary malignancies are relatively uncommon malignancies that generally have a poor prognosis (Figure 1). In 2012, almost 42,000 new cases of pancreatic cancer and 10,000 new cases of gallbladder and bile duct cancer were expected in the USA ^1^. The prognosis of patients with pancreatic cancer and intrahepatic cholangiocarcinoma is poor, with an estimated 5-year overall survival of 2–5%. Patients with extrahepatic bile duct cancer and gallbladder cancer have a slightly better survival, but the overall 5-year survival is still only 12–15% ^2^. Worldwide, the mortality rates for bile duct cancer seem to have decreased slightly over recent decades, a trend that may in part be due to improved diagnostic modalities and more widespread use of the surgical removal of the gallbladder (cholecystectomy) for gallstones (these being a known cause of gallbladder cancer) ^3^. Despite the observed improvements in prognosis, the majority of patients with pancreatobiliary carcinoma still present at an advanced stage where resection is not feasible ^2^. Of all patients with newly diagnosed pancreatic cancer, almost half have metastatic disease at diagnosis, with an additional 22% having either node-positive disease or a large tumor invading adjacent organs (known as a T4 lesion) ^2^. Bile duct carcinomas tend to be less advanced at presentation than pancreatic cancer, which probably explains the better prognosis to some extent. Other factors, such as differences in the genetic basis of these cancers, may provide further insight into the differences in outcomes. Further therapy following resection (adjuvant therapy) has been shown to improve the outcome of patients with pancreatic cancer. The best studied adjuvant therapies are systemic therapy for 6 months with gemcitabine and post-operative concurrent chemotherapy with gemcitabine and 5-fluorouracil but the optimal adjuvant therapy remains undefined. Although adjuvant chemotherapy or chemoradiotherapy for resected pancreatic cancer has been shown to be beneficial, most patients who undergo resection eventually succumb to the disease ^4– 6^. The role of adjuvant therapy for resected bile duct cancer is less certain and there is a dearth of well-conducted prospective studies on the subject. A recent phase III trial did not show conclusive evidence for the benefit of adjuvant chemotherapy following resection of periampullary adenocarcinoma ^7^. After adjusting for other prognostic factors, a benefit of adjuvant therapy was observed. Multiple retrospective studies do, however, support the role of radiotherapy or chemoradiotherapy, although the benefits seem modest ^8– 11^. Two recent meta-analyses have also suggested that there may be benefit of adjuvant therapy ^12, 13^. The majority of patients will at some point be diagnosed with advanced disease, either at the time of first diagnosis or at a later stage once the cancer recurs. There is thus a great need for improvements in advanced therapy for these malignancies. This article will discuss palliative treatment options for pancreatobiliary malignancies from the standpoint of medical and radiation oncology, focusing on chemotherapy, radiotherapy or both. A discussion of the treatment of the symptoms of advanced pancreatobiliary malignancies such as pain management and treatment of biliary obstruction is outside the scope of this review ^14, 15^.

## Pancreatic carcinoma

### Locally advanced (unresectable) pancreatic carcinoma

Many patients with pancreatic cancer present with unresectable cancer and, in fact, only 10–20% of patients are deemed to be operative candidates ^16^. For the remainder of patients, the outcome is bleak, with nearly all patients succumbing to their disease within 2 years of diagnosis. Patients with advanced locoregional (i.e. localized, nonmetastatic) disease have a median survival of 9–10 months, which is only a few months better than in patients with metastatic disease ^17^. The optimal therapy for locally advanced pancreatic cancer is not known, but chemotherapy, radiation therapy and a combination thereof is frequently used. A small randomized trial reported improved survival and better quality of life (QOL) in patients treated with a combination of the DNA synthesis inhibitor 5-fluorouracil (5-FU) and radiation therapy ^18^. Chemotherapy alone has also been shown to improve survival in patients with advanced pancreatic cancer when compared with the best supportive care ^19^. Two studies evaluating the benefits of adding chemotherapy to radiation therapy yielded conflicting results, but a pooled analysis suggested a benefit from concurrent chemoradiotherapy compared with radiotherapy alone ^20– 22^. Two recent trials compared chemoradiotherapy with chemotherapy alone and came to a different conclusion, with one suggesting a benefit of adding radiotherapy and the other not ^23, 24^. The use of concurrent chemoradiation therapy for locally advanced pancreatic cancer is also supported by phase II studies ^25, 26^. Chemoradiotherapy was not found to be superior to chemotherapy alone in a recent systematic review, but the heterogeneity and small size of the included studies makes comparisons difficult ^27^. It is worth mentioning that the prematurely closed Eastern Cooperative Oncology Group (ECOG) study 4201, which compared gemcitabine monotherapy with chemoradiation therapy using gemcitabine as a radiosensitizer followed by gemcitabine monotherapy, suggested a modest benefit of the combination therapy ^24^. It seems that not all patients may benefit from the addition of radiotherapy, and the challenge is how best to identify those who may be helped with combination therapy.

An increasingly used approach is to initiate chemotherapy (induction therapy), and if there is no evidence of progression with new liver metastases after 2–3 months as visualized by CT scanning, patients are considered for concurrent chemoradiotherapy. The rationale for this approach is that a substantial proportion of patients will progress within this timeframe while on chemotherapy and the site of progression is frequently in the liver or elsewhere outside of the conventional radiation field. These patients would probably not have benefited from the addition of radiotherapy. It is likely that induction therapy selects out those patients who would be more likely to benefit from the addition of radiotherapy. This approach is supported by two recent retrospective studies and is increasingly being used in the USA ^28, 29^.

In summary, concurrent chemoradiation either upfront or preceded by 2–3 months of chemotherapy seems to be an appropriate standard for the management of locally advanced pancreatic cancer. The role of chemotherapy alone without radiation is less certain – but certainly a viable option for patients who either choose not to receive radiation therapy or have a contra-indication to such therapy. Recent studies have shown that complete loss of expression of the signal transduction protein SMAD4 is associated with a higher incidence of distant metastases and that tumors that retain SMAD4 expression are less likely to metastasize ^30, 31^. Determination of SMAD4 expression may have a role in guiding therapy, in which patients with tumors expressing SMAD4 could be considered for incorporation of locoregional therapy into the treatment plan.

### Metastatic pancreatic carcinoma

**Figure 2.**
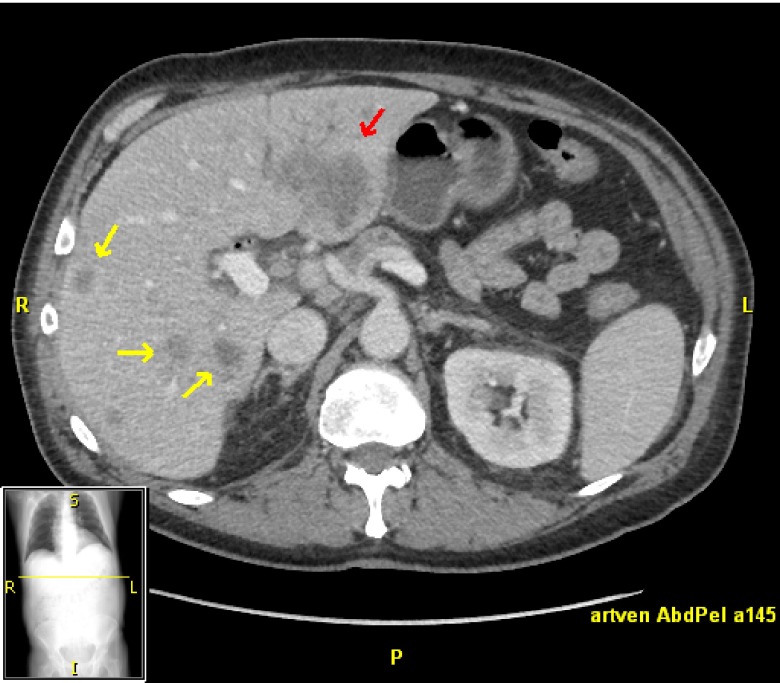
Metastatic pancreatic cancer. Multiple small liver metastases (yellow arrows) and a larger metastasis in the left lobe of the liver (red arrow).

Metastatic pancreatic carcinoma is a uniformly fatal disease, and systemic chemotherapy is only modestly effective in prolonging survival and maintaining quality of life. Pancreatic cancer most commonly metastasizes to the liver (Figure 2). The results of several key phase III trials in first-line therapy for pancreatic cancer are reported in Table 1. Gemcitabine, which is a nucleoside analog, either alone or in combination, has been the mainstay of therapy for more than a decade. Gemcitabine was reported to be more effective than 5-fluorouracil in a landmark trial published in 1997 that established gemcitabine as the chemotherapeutic agent of choice for advanced pancreatic cancer ^32^. Although gemcitabine only modestly prolonged survival (median survival 5.65 vs. 4.41 months), the effect on the clinical benefit response, a composite of measurements of pain, performance status, and weight loss was more marked. Since then, numerous trials have explored the addition of other drugs in combination with gemcitabine, generally with unimpressive results. Individual trials and meta-analyses have shown benefits of gemcitabine-based combinations over gemcitabine monotherapy, but the magnitude in terms of clinical improvements is small and in some cases of questionable clinical significance ^19, 33^. The benefits from combination therapy may be more pronounced in patients with good performance status ^33, 34^. Performance status reflects the physical activity of patients and their ability to care for themselves and is commonly graded on a scale of 0 to 4 (the Eastern Cooperative Oncology Group scale) where patients with a performance score of 0 have no restrictions from their malignancy and patients with a score of 4 are completely disabled, bedbound and unable to carry out any self care. Gemcitabine monotherapy remains an acceptable treatment option, especially for patients with impaired performance status.

**Table 1.** Key randomized phase III trials of first-line chemotherapy for metastatic pancreatic cancer.

Author/Year	Treatment	Progression-Free survival (months)	Overall survival (months)	12 month overall survival (%)
Burris *et al*. 1997	Gemcitabine	2.33*	5.65*	18
	5-fluorouracil	0.92	4.41	2
Moore *et al*. 2007	Gemcitabine + erlotinib	3.55*	6.24*	23*
	Gemcitabine	3.75	5.91	17
Cunningham *et al*. 2009	Gemcitabine + capecitabine	5.3*	7.1	24.3
	Gemcitabine	3.8	6.2	22
Conroy *et al*. 2011	FOLFIRINOX	6.4*	11.1*	48.4
	Gemcitabine	3.3	6.8	20.6
Von Hoff *et al*. 2013	Gemcitabine + nab-paclitaxel	5.5*	8.5*	35*
	Gemcitabine	3.7	6.7	22

*Statistically significant with p<0.05

Two commonly used agents added to gemcitabine are capecitabine and erlotinib. Capecitabine, an oral fluoropyrimidine, when added to gemcitabine (GEM-CAP) was shown to improve progression-free survival with a nonsignificant trend towards improvement in overall survival compared with use of gemcitabine alone in a meta-analysis and is a reasonable combination for patients with good performance status ^35^. As ~90% of pancreatic cancers have an activating mutation in the GTPase KRAS protein ^36^, significant effort has been put towards targeting the RAS–RAF–MEK–ERK pathway along with other pathways. The addition of erlotinib, an inhibitor of the epidermal growth factor receptor (EGFR), to gemcitabine, resulted in a minimal but statistically significant improvement in overall survival by 2 weeks (median overall survival 6.2 vs. 5.9 months), although at the cost of increased toxicity ^37^. The observed survival increase with the addition of erlotinib is of questionable clinical significance, but the trial is remarkable for the fact that it is the first and only phase III trial to show a benefit from adding a targeted agent to gemcitabine. The gemcitabine–erlotinib combination was subsequently approved for use in the USA but not in Europe. Other recent studies did not, however, show any benefit of adding bevacizumab, sorafenib, axitinib or cetuximab to gemcitabine ^38– 41^. Farnesyltransferase inhibitors targeting the Ras pathway have not proven to be successful in management either ^42^. A recent phase I/II study of gemcitabine combined with the mitotic inhibitor nab-paclitaxel yielded promising results, where patients with increased levels of stromal ‘secreted protein acidic and rich in cysteine’ (SPARC) had a greater degree of benefit compared with those patients who had lower stromal SPARC (overall survival of 17.8 vs. 8.1 months, P=0.0431) ^43^. The results of a larger phase III trial comparing this combination with gemcitabine monotherapy were presented at the Gastrointestinal Cancers Symposium in January 2013 ^44^. In this trial 861 patients were randomized and received either weekly nab-paclitaxel with gemcitabine or gemcitabine alone. Overall response rates (23% vs. 7%), progression-free survival, PFS (5.5 vs. 3.7 months) and overall survival, OS (8.5 vs. 6.7 months) were all significantly improved in the combination arm. Grade 3 or more adverse events more commonly seen in the combination arm included neutropenia (38% vs. 27%), fatigue (17% vs. 7%) and neuropathy (17% vs. 1%) but overall the combination was well tolerated. This combination is therefore likely to get approval by the FDA in the coming months. The role of SPARC in patient selection will be further elucidated from the phase III data. The results of a phase III trial with the combination of masitinib (multityrosine kinase inhibitor) and gemcitabine are also expected in 2013, where, according to a press release from the pharmaceutical company AB Science, two subgroup populations had increased overall survival by 6 and 2.7 months, characterized by a genetic biomarker and patients with cancer pain, but not in the overall patient population ( http://www.ab-science.com/file_bdd/1351622639_abscienceresultph3pancreasvdefuk.pdf).

The microenvironment within pancreatic cancers is frequently hypoxic relative to normal pancreatic tissue and the hypoxic properties of pancreatic cancers are being exploited in clinical trials ^45^. A recent randomized phase II study of the hypoxia-targeted prodrug TH-302 with gemcitabine in previously untreated patients showed an improvement in progression-free survival (PFS) with the combination when compared with gemcitabine alone ^46^. These results will need to be confirmed in a larger trial.

Whole-genome exome sequencing of pancreatic adenocarcinomas has recently been completed and should equip us with more drug targets in the future ^47, 48^.

The role of gemcitabine as an essential component of the chemotherapy for pancreatic cancer has recently been called into question. A phase III trial compared a multi-agent regimen of 5-fluorouracil, leucovorin, irinotecan and the DNA synthesis inhibitor oxaliplatin (FOLFIRINOX) with gemcitabine alone. Overall survival was markedly improved in the FOLFIRINOX group compared with the gemcitabine group. The median overall survival of patients receiving FOLFIRINOX was 11.1 months vs. 6.8 in the gemcitabine group. FOLFIRINOX was more likely to result in adverse events, and febrile neutropenia was seen in 5.4% of patients. Despite higher toxicity, fewer patients in the FOLFIRINOX group had deterioration of their quality of life at 6 months (66% vs. 31%). Fewer than 40% of patients in the trial had tumors located in the head of the pancreas compared with 60–70% of all patients presenting with pancreas cancer. It is therefore unclear whether the results are applicable to most patients with pancreatic cancer, and biliary obstruction with jaundice would certainly preclude giving irinotecan in many cases. However, a subgroup analysis indicated a similar benefit to patients with tumors outside the head of the pancreas. Subgroup analyses also showed that patients older than 65 years and patients with an ECOG performance status of 1 also benefited from more aggressive therapy. Furthermore, patients receiving FOLFIRINOX were less likely to experience a decline in quality of life compared with patients on gemcitabine. The encouraging results have led to the acceptance of FOLFIRINOX chemotherapy for patients with metastatic pancreatic cancer, especially those with good performance status.

The prognosis for patients progressing after first-line (i.e. initial) therapy is very poor, and no standard treatment approach exists. These patients should be considered for enrollment on clinical trials whenever possible. In patients previously treated with gemcitabine, subsequent second-line therapy with oxaliplatin, 5-fluorouracil and leucovorin (OFF) has been shown to improve overall survival modestly when compared with best supportive care alone ^49^. FOLFIRINOX may be an option for younger patients with good performance status who have not received such therapy previously, but prospective studies are lacking ^50^. Other agents such as taxanes and irinotecan may have activity in pretreated patients and can be considered in selected patients ^51– 53^. No prospective data exist regarding therapy for patients progressing after first-line FOLFIRINOX, but gemcitabine, either alone or in combination with other agents such as capecitabine or nab-paclitaxel may be used if performance status allows.

## Biliary carcinoma (intrahepatic and extrahepatic cholangiocarcinoma and gallbladder carcinoma)

### Locally advanced (unresectable) biliary carcinoma

Carcinomas of the biliary system are uncommon malignancies and frequently unresectable at the time of diagnosis ^54^. Given the relative rarity of these cancers, very few large treatment trials have been performed and much of the evidence guiding treatment decisions stems from retrospective and epidemiological studies.

Patients with locally advanced biliary carcinoma may benefit from concurrent chemoradiation therapy for palliative purposes, and such treatment is currently suggested as one of the treatment options in published guidelines ^55, 56^. Fluoropyrimidines such as 5-fluorouracil or capecitabine are frequently used as radiosensitizing agents. Other locoregional treatment options for locally advanced but unresectable bile duct cancer include radiation brachytherapy (in which the radiation source is placed at very close proximity within the tumor) and photodynamic therapy (in which light and photosensitizing agents are used to kill the cancer cells) ^57^. Despite aggressive local therapy, locoregional failures are frequent ^58^. For those patients who do not desire to have radiation therapy or where such therapy is contraindicated, chemotherapy alone is recommended. Patients with intrahepatic cholangiocarcinoma limited to the liver may be considered for liver-directed therapy such as hepatic artery chemoembolization or radioembolization ^59, 60^. Best supportive care remains an option for patients with poor performance status and for patients who do not want any anti-cancer therapy. Such patients may be significantly helped using procedures aimed at improving or preserving quality of life, including biliary drainage and aggressive pain and symptom control.

### Metastatic biliary carcinoma

Systemic therapy (chemotherapy) is frequently used in the management of metastatic biliary carcinoma, but again there is a dearth of well-conducted randomized trials owing to the relative rarity of this malignancy. A small study of patients with either pancreatic cancer or biliary cancer showed that chemotherapy resulted in improved survival and quality of life compared with using no such therapy ^61^. A similar study showed that the combination of gemcitabine and oxaliplatin (GEMOX) was superior to best supportive care in terms of overall and progression-free survival in patients with advanced carcinoma of the gall bladder ^62^. Gemcitabine, either alone or in combination with other chemotherapeutic agents, is commonly used for patients who wish to receive chemotherapy, but multiple other regimens not containing gemcitabine do exist ^63– 70^. Gemcitabine has been shown to be both safe and effective, even in elderly patients ^65^. Gemcitabine has been used in combination with platinum agents and fluoropyrimidines in multiple small phase II trials, but it was not until 2009 that a regimen that can be considered a standard emerged. The ABC-02 trial was a multicenter randomized phase III trial conducted in the United Kingdom that compared gemcitabine monotherapy with gemcitabine combined with low-dose cisplatin ^71^. In this trial, 410 patients with locally advanced or metastatic biliary cancer were randomized to receive either cisplatin (25 mg/m ^2^) with gemcitabine (1000 mg/m ^2^) on days 1 and 8, every 3 weeks for eight cycles or gemcitabine alone (1000 mg/m ^2^) on days 1, 8, and 15, every 4 weeks for six cycles for up to 24 weeks. Patients on the combination therapy arm had both longer overall survival (11.7 vs. 8.1 months) and progression-free survival (8 vs. 5 months). Both regimens had acceptable toxicities. The results from a smaller Japanese phase III study support the superiority of the gemcitabine–cisplatin combination compared with gemcitabine alone ^70^. The combination of gemcitabine and cisplatin can thus be considered a reasonable standard for first-line therapy of metastatic biliary cancer in patients with good performance.

Trials evaluating targeted agents (such as key receptors and signaling proteins) in advanced hepatobiliary carcinoma have largely been disappointing and, to this date, no clear role for such therapy exists.

Vascular endothelial growth factor (VEGF, a signaling protein that regulates blood vessel growth) has been found to be overexpressed in cholangiocarcinoma as well as many other tumors ^72^. Other mutations such as activating mutations in BRAF (22%) and KRAS (45%) have been reported ^73^, and these findings have led to several phase I/II clinical trials with targeted agents. The angiogenesis inhibitor bevacizumab was given with the EGFR inhibitor erlotinib in a phase II trial where 9 of 53 patients had partial responses but only 6 (12%) were sustained, and the duration of best response was similar to that seen with chemotherapy (average of 7.6 months) ^74^. A phase II trial where bevacizumab was given with GEMOX found it to be safe and effective (41% with partial response) ^75^. GEMOX paired with cetuximab in a phase II trial produced similar results (63% with objective response) ^76^.

Despite promising phase II trials, targeted agents have not produced any breakthrough results, and phase III trials are needed to evaluate whether combinations including targeted drugs are superior to standard chemotherapy. GEMOX with and without erlotinib was compared in a phase II trial and, although erlotinib did improve response rates (30% vs. 16%), it did not significantly affect progression-free survival (median 5.8 vs. 2 months), which was the primary endpoint ^73^. In an unplanned analysis of cholangiocarcinomas only (the trial included gallbladder and ampullary cancers as well), the progression-free survival was significantly better in the erlotinib group (5.9 vs. 3 months), but the erlotinib treatment had more toxicities ^77^. Other targeted agents that have been evaluated in advanced biliary cancers include the tyrosine kinase inhibitor sunitinib and the MEK 1/2 (MAP kinase kinase 1/2) inhibitor selumetinib ^78, 79^. The role of the newer agents needs to be defined in larger trials.

Second-line therapy is commonly offered to patients progressing after initial chemotherapy, but the data supporting such therapy are very limited. A recent retrospective study suggested that there are modest benefits of second-line therapy, with a possible advantage of doublet therapy (in which two agents are co-administered) compared with single-agent therapy ^80^. It is reasonable to offer patients with preserved performance status a second-line systemic therapy, and an attempt should be made to choose agents from a class different from that used in the first-line setting given that the cancer cells may have become resistant to the previously used class of drugs. All such patients should be considered for participation in a clinical trial if available.

## Concluding remarks

Advanced pancreatic and biliary cancer remain difficult to treat, and responses are usually short-lived and the prognosis poor. Combination therapies for metastatic pancreatic cancer such as using the DNA synthesis inhibitors FOLFIRINOX or gemcitabine with the cell-division inhibitor nab-paclitaxel appears more effective than gemcitabine alone, but single-agent gemcitabine remains an appropriate option for the elderly and for patients with impaired performance status. Second-line therapy for advanced pancreatic cancers is not very effective, and better treatment options are clearly needed. Using gemcitabine and cisplatin is a reasonable first-line therapy for advanced biliary cancer, but, as with pancreatic cancer, second-line therapy has yielded disappointing results, and the prognosis remains poor. It is unlikely that further refinements of cytotoxic chemotherapy regimens will result in substantial improvement of prognosis. There is an unmet need for better treatment options and future improvements are likely going to be secondary to new targeted agents and not cytotoxic chemotherapy.
