# Supplementary material for: Advances in systemic therapy for advanced pancreatobiliary malignancies
Source: F1000Res. 2013 Apr 8;2:105. [Version 1] doi: 10.12688/f1000research.2-105.v1 (PMC3752657; doi:10.12688/f1000research.2-105.v1)
Supplement: Peer review history

# Peer review: Referee response for version 1

**Published:** 2013-04-22

**Authors:** Christina Wu

**Affiliations:** Division of Medical Oncology, Ohio State University, Columbus, OH, USA

The title and abstract are concise and appropriate for the content. The abstract summarizes the paper well. The review covers the phase II and III clinical trials for advanced hepatobiliary cancers. The conclusions are well balanced and support the findings of the paper.

I have read this submission. I believe that I have an appropriate level of expertise to confirm that it is of an acceptable scientific standard.

# Peer review: Referee response for version 1

**Published:** 2013-04-15

**Authors:** Madappa Kundranda

**Affiliations:** Cancer Treatment Centers of America, Goodyear, AZ, USA

**Title and Abstract:** Succinct. 

**Content:** Very good compilation of the latest and most appropriate studies including the recently presented MPACT study in advanced pancreatic cancer.

**Conclusions:** Balanced.

I have read this submission. I believe that I have an appropriate level of expertise to confirm that it is of an acceptable scientific standard.
